# Effects of antioxidants on physicochemical properties and odorants in heat processed beef flavor and their antioxidant activity under different storage conditions

**DOI:** 10.3389/fnut.2022.966697

**Published:** 2022-08-30

**Authors:** Zeyu Zhang, Fanyu Meng, Bei Wang, Yanping Cao

**Affiliations:** Beijing Advanced Innovation Center for Food Nutrition and Human Health, School of Food and Health, Beijing Higher Institution Engineering Research Center of Food Additives and Ingredients, Beijing Technology and Business University, Beijing, China

**Keywords:** heat processed beef flavor, antioxidant, odorant, physicochemical, lipid oxidation

## Abstract

Heat processed beef flavor (HPBF) is a common thermal process flavoring, whose flavor properties can be affected by lipid oxidation during storage. Addition of antioxidants is an option to avoid the changes of HPBF induced by lipid oxidation. In this study, the effects of three antioxidants, tert-butylhydroquinone (TBHQ), tea polyphenol (TP), and L-ascorbyl palmitate (L-AP), on volatile components, physicochemical properties, and antioxidant activities of HPBF were studied over 168 days at different temperatures (4, 20, and 50°C). Although all three antioxidants had little effect on browning, acidity, water activity, and secondary lipid oxidation products, L-AP and TBHQ showed greater capabilities to prevent the formation of primary lipid oxidation products than TP. According to the results of oxidation reduction potential and DPPH radical scavenging experiments, TBHQ had better antioxidant ability compared to L-AP and TP during the storage. Of note, TBHQ affected the flavor profiles of HPBF, mainly on volatile odorants produced by lipid degradation. TBHQ could mitigate the development of unfavorable odorants. This study indicated TBHQ would enhance lipid oxidation stability and maintain physicochemical properties and flavor profiles of HPBF during storage. It suggested that TBHQ could be applied as an alternative additive to improve the quality of HPBF related thermal process flavorings.

## Introduction

Heat processed beef flavor (HPBF) is known as one of the “thermal process” flavorings produced by heating a mixture of two or more precursor materials (1). A major purpose of “thermal process” flavorings is to enhance the characteristic meaty note of foods (2, 3). However, the negative changes in quality during storage might be affected by storage time and temperature. It is well-known that the Maillard reaction and lipid oxidation are of the utmost importance for the development of meaty note, which occur as the main processes during thermal treatment and storage (4). Lipids determine the flavor properties of products with a meaty note (5, 6). Lipid oxidation products give generally fatty and meaty notes which determines the aroma differences between meats from different species (7). Lipid-derived compounds, such as certain aldehydes with higher odor detection threshold values, generally have a higher contribution to overall favor profiles than the sulfur or nitrogen heterocyclic compounds formed through the Maillard reaction (5, 8).

The quality changes caused by lipid oxidation reactions are more prominent than those caused by reactions with other precursors (9, 10). All lipid-containing products, even those with minimal unsaturated fatty acid contents, have essentially the potential ability to undergo lipid oxidation in highly processing or during prolonged storage (11). Fatty acids presented in animal meats, especially polyunsaturated fatty acids increase the risk of oxidation reaction, leading to undesirable flavors (12). Radical species produced by lipid oxidation may have an unfavorable effect on the physicochemical properties, such as browning (13). Thus, appropriate evaluation and control of lipid oxidation are controversial issues.

Antioxidants have been employed to improve food quality by preventing lipid oxidation (14). Tert-butylhydroquinone (TBHQ), L-ascorbyl palmitate (L-AP), and tea polyphenol (TP) are the most commonly used antioxidants in the current relevant Chinese national standard GB-2760 (15). These antioxidants have the potential ability to prevent lipid oxidation in meat-based products and maintain the flavor stability of food (16). Lipid-soluble TBHQ as one of the synthetic antioxidants has been widely utilized to prevent lipid oxidation (17). Even the addition of TP, a water-soluble antioxidant, could inhibit the lipid oxidation of sausages made of meat (18), which has shown antioxidant performance comparable to TBHQ (19). L-AP as one of the intermediate polarity antioxidants provided antioxidant protection comparable to TBHQ during a long-term storage of flaxseed oil (20).

However, the effects of antioxidants with different solubility on the performance and flavor properties of the water-oil mixtures are controversial (21, 22). Frankel et al. (23) reported that the antioxidant activity of TBHQ was higher than that of L-AP in the emulsion system, which could mitigate flavor deterioration. This finding by Frankel et al. (23) is consistent with the results obtained by Gordon and Kourkimskå (24) in deep-fried rapeseed oil (as with bulk oils). This could be due to the fact that lipophilic antioxidants are more potent in emulsions than in bulk oils (considered water-in-oil nanoemulsions) (25). In contrast, Wanasundara and Shahidi (26) found that TBHQ and butylated hydroxyanisole (BHA) were not as effective as catechins (the primary component of TP) in preventing the oxidation of seal blubber or menhaden oil, as well as the odor and flavor of lipid-containing foods. Zhang et al. (27) reported that polyphenolic antioxidants (e.g., TP) can effectively alleviate lipid oxidation while also enhancing unpleasant odors due to their astringency. Consequently, it is still challengeable to control lipid oxidation and improve aroma profiles in parallel.

Hence, it is necessary to obtain the optimum antioxidant to control lipid oxidation and mitigate flavor deterioration of HPBF within a water-oil mixture during the storage. To achieve this purpose, TBHQ, TP, and L-AP were added into HPBF as lipid soluble, water soluble, and intermediate polarity antioxidants respectively. A potential antioxidant was determined by evaluating the effects of three antioxidants on lipid oxidative stability, physicochemical properties, and volatile components of HPBF. The purpose of this study was to provide a basis for the development of thermal process flavorings with high quality.

## Materials and methods

### Materials and reagents

Amino acids (glycine, cysteine), monosaccharide (glucose, xylose), Protamex (120 U/mg), and Flavourzyme (20 U/mg) were obtained from Shanghai Yuanye Biotechnology Co., Ltd. (Shanghai, China). Yeast extract was purchased by Beijing Aoboxing Biotech Co., Ltd. (Beijing, China). Spices (including clove, cinnamon, and fennel), fresh beef lean meat, fresh onion, garlic, and ginger were purchased from the local supermarket.

Reagents used in the study are listed as follows: isooctane, isopropyl alcohol, potassium thiocyanate, ferrous chloride anhydrous, ethanol, and butanol were purchased from Fuchen Chemical Reagent Co., Ltd. (Tianjin, China). Trichloroacetic acid (TCA) and 2-thiobarbituric acid (TBA, biochemical reagent, purity ≥ 98.5%) were obtained from Sinopharm Chemical Reagent Co., Ltd. (Beijing, China). Ethylene diamine tetraacetic acid (EDTA) was provided by Beijing Biotopped Science and Technology Co., Ltd. (Beijing, China). 1,1-Diphenyl-2-picrylhydrazyl (DPPH) was purchased from Shanghai Yuanye Biotechnology Co., Ltd. (Shanghai, China). Tert-butylhydroquinone (TBHQ), tea polyphenols (TP), L-ascorbyl palmitate (L-AP), and 1,1,3,3-tetraethoxy propane were purchased from Shanghai Macklin Biochemistry Co., Ltd. (Shanghai, China). 2-Methyl-3-heptanone and n-alkane were purchased by Sigma-Aldrich (Shanghai, China). Methanol [purity ≥ 99.9%, Mreda Technology Inc. (United States)], and ammonium acetate (purity ≥ 98.7%, Thermo Fisher Scientific, Inc.) were of HPLC grade. In addition, n-hexane (purity ≥ 99.9%) of GC grade was purchased from Mreda Technology Inc. (United States). Distilled-deionized water used in all experiments was purified using a Milli-Q Gradient (Millipore, Bedford, MA, United States). Helium gas (99.9992% purity) was provided by Beijing Tianlirenhe Material Trade Co. Ltd. (Beijing, China).

### Samples preparation

The HPBF samples (control) was prepared by mixing of an enzymatic hydrolysate and other ingredients. To prepare the enzymatic hydrolysate, the fresh beef lean meat was trimmed of its fat and connective tissue and then crushed prior to the experiment. The meat paste was hydrolyzed by 0.21 g of Protamex (120 U/mg) and 0.42 g of Flavourzyme (20 U/mg) for 60 and 240 min, respectively. After cooling of the above enzymatic hydrolysate, the ingredients of HPBF were weighed according to the proportion of ingredients including 21.0% bovine bone, 2.7% yeast extract, 0.3% glycine, 0.3% cysteine, 0.9% glucose, 0.3% xylose, 4.8% fresh onion, 1.9% garlic, 0.9% ginger, and 0.076% spice powder. Those ingredients of HPBF were completely mixed with the enzymatic hydrolysate before being placed in a steam sterilization pot at 115°C for 60 min. In the treatment group, TBHQ, TP, and L-AP were added at a level of 0.02% based on fat content.

The sterilized HPBF was filtered and filled into 40 mL glass vials (Supelco, United States) sealed with screwed top PTFE/silicone septa. The HPBF samples were stored at 4, 20 and 50°C, respectively. Among them, 4°C represents low temperature and 20°C represents room temperature. In addition to accelerate the storage, the temperature of 50°C also applied and represents the high temperature conditions. The relevant physicochemical characteristics and odorants were determined for 168 days including a total of 17 sets (1, 7, 14, 21, 28, and so on up to 168 days).

### Oxidative stability

#### Oxidation reduction potential

Oxidation reduction potential (ORP) was measured using a calibrated hand-held ORP Electrode LE501 [Mettler Toledo International Trading (Shanghai) Co., Ltd., Shanghai, China]. The ORP readings (mV) was determined by the method described in Capuani et al. (28) with minor modifications. The redox electrode was inserted into the HPBF immediately after leaving the electrolyte solution of 3 M KCl in order to minimize the effect of air. The redox electrode was checked using a redox buffer solution of 220 mV/pH 7 from Mettler Toledo before each use.

#### Peroxide value

Lipid peroxides were quantitated using a modified method by Chaiyasit et al. (29). The original sample (1 mL) and 5 mL of isooctane-isopropyl alcohol (2:1, v/v) solution were stirred by eddy current for 30 s, and then centrifugation at 9000 rpm for 5 min. The clear upper layer (2 mL) was mixed thoroughly with 20 μL of potassium thiocyanate solution and 20 μL of ferrous chloride solution (0.144 M). The mixture was mixed with butanol-methanol (v/v, 2:1) solution to obtain a 5 mL mixed solution. Absorbance was measured at 510 nm after incubation away from light for 20 min at room temperature. A standard curve made up of diluted ferrous chloride solutions was used to calculate lipid peroxide concentrations in the HPBF. The results of PV value were expressed as the uptake of mEq of active oxygen per kg of lipid (mEq/kg).

#### Thiobarbituric acid reactive substances

The TBARS assay was performed according to a previously method described by Papastergiadis et al. (30) with slight adjustments. Briefly, 1 g HPBF was mixed with a total of 5 mL of 7.5% TCA (w/v) with 0.02% (w/v) of EDTA. The mixed solution was homogenized with a refrigerated centrifuge (Hunan Herexi Instrument and Equipment Co., Ltd., Changsha, China) for 10 min at 8000 rpm. The homogenate was treated with 5 mL TBA reagent (20 mM) at 90°C for 30 min, and then filtered through a 0.22 Millipore membrane filter (MREDA Technology Inc., Beijing, China) after cooled using running water. Ten μL mixture solution was separated by Waters 2695 HPLC on a Waters SunFireTM C18 column (4.6 × 250 mm, 5 μm) using a mobile phase of 10 mM ammonium acetate and methanol (7:3, v/v). The column temperature was 30°C. The detection wavelength was 532 nm using Waters 2996 photodiode array detector (PAD). A standard curve made up of diluted 1,1,3,3-tetraethoxy propane solutions was used to calculate the amount of TBARS. The results were given in milligrams of MDA equivalents per kilogram of the sample (mg MAD/kg).

### 1,1-Diphenyl-2-picrylhydrazyl radical scavenging activity

The antioxidant activity in the DPPH assay was performed in the manner of the descriptions given by previous reports (31) with minor modifications. A dilution of 1 mL HPBF (diluted 500 times with deionized water) was thoroughly mixed with 3 mL of DPPH ethanol solution (0.1 mM). The mixture was incubated at room temperature for 30 min in the dark. The ethanol control group was added as a blank for DPPH. An ultraviolet-visible spectrophotometer (UV-1240, Shimadzu, Co., Ltd., Tokyo, Japan) was used to measure the absorbance at 517 nm to estimate the radical scavenging capacity of antioxidant samples at each storage time.

### Physicochemical characteristics evaluation

#### Browning determination

Browning degree was linked with the absorbance at 420 nm (32). Non-enzymatic browning was monitored using the ultraviolet-visible spectrophotometer (UV-1240, Shimadzu, Co., Ltd., Tokyo, Japan) against water at 420 nm.

#### pH level determination

The pH was measured by using a calibrated hand-held pH meter [TB-214, Mettler Toledo International Trading (Shanghai) Co., Ltd., Shanghai, China]. The pH was calibrated using pH 4, pH 6.86, and pH 9.18 calibration buffers.

#### Water activity (a_w_) determination

The water activity (a_w_) was measured using an AquaLab 4TEV water activity meter in capacitance mode after samples reached equilibrium at 25°C.

### Volatile compounds analysis

#### Extraction of volatiles by HS-SPME

Volatile compounds of HPBF were analyzed by means of solid-phase microextraction-Gas chromatography/Mass spectrometry (SPME-GC/MS) with a DB-WAX column (30 m, 0.25 mm i.d., 0.25 μm film thickness, Agilent). For each sample, 10 mL of HPBF were added to a 40 mL headspace bottle (Supelco, United States) containing 1 μL of 2-methyl-3-heptanone (diluted by a factor of 1000 in hexane) as the internal standard, and allowed to stand for 30 min at 45°C. The DVB/carboxen/PDMS fiber (Supelco, United States) was then exposed to the headspace of HPBF for 30 min while the vial was maintained at 45°C, and desorbed at 250°C for 5 min in the injection port after headspace collection. The experiments were performed in triplicate.

#### GC-MS analysis

GC-MS analyses were performed on an Agilent 7890B gas chromatography coupled to an Agilent 5977A mass spectrometer. The DVB/carboxen/PDMS fiber was desorbed in splitless mode with a splitless inlet liner of 0.75 mm inlet diameter (Agilent) suitable at 250°C for SPME analysis. The mass spectrometer was operated in electron impact mode with the electron energy set at 70 eV to obtain the mass spectra. A mass scan from *m/z* 35 to *m/z* 400 was performed with the ion source at 230°C. Helium was used as the carrier gas at a rate of 1 mL/min. The oven program started at 40°C for 2 min, then increased at 4°C/min to 190°C, and subsequently to 240°C at 8°C/min.

#### Isolation and identification of the volatiles

Volatile compounds were identified by comparing mass spectrometry patterns from the NIST14 database with linear retention indices (RI) based on a homologous series of even numbered n-alkanes (C7-C40). A semi-quantitative analysis of the detected volatile compounds was performed based upon comparison of their GC-MS peak regions to the internal standard (2-methyl-3-heptanone).

An Agilent 7890B gas chromatography coupled to a sniffing port ODP-3 from Gerstel was employed to analyze HPBF under the same analytical conditions. Volatile odorants were sniffed by trained panelists (two females and one male) on the top of the sniffing port and were used for the PCA analysis.

### Statistical analysis

The least significant difference among different samples was analyzed using the one-way analysis of variance (ANOVA) with SPSS Statistics 22.0 (IBM Corporation, New York, NY, United States). All analyses for significant differences set at a 5% significance level. Statistical analysis each experiment was independently triplicated to derive an average and standard deviation. The data were subjected to principal component analysis (PCA) and illustrated using Origin 2021b (OriginLab Corporation, Northampton, MA, United States).

## Results and discussion

### Oxidative stability analysis

#### Oxidation reduction potential

Oxidation reduction potential is a measure of a chemical species to acquire electrons and thereby be reduced. As shown in Supplementary Figures 1A–C, the ORP values increased steadily in all treatments (TBHQ, TP, and L-AP) with the increasing storage time. The results showed that lipids in HPBF were continuously oxidized during storage. Besides, three antioxidants (TBHQ, TP, and L-AP) had only a small effect on the potential redox of HPBF during storage. There was no significant difference (*p* > 0.05) between the treatment groups (TP or L-AP) and control. An addition of TBHQ in HPBF decreased potential redox values after 98 days compared to the control at a lower temperature (4°C). This suggested that the antioxidant effect of TBHQ in HPBF was superior to that of TP and L-AP at 4°C. As a results, TBHQ may be a potent antioxidant that produce an additive protective effect during storage, inhibiting the oxidative degradation of lipids.

#### Peroxide value

**FIGURE 1:**
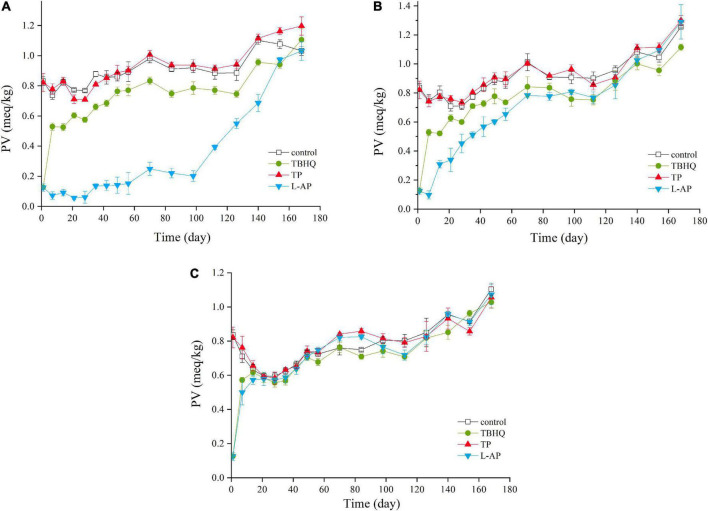
PV values of HPBF with the addition of TBHQ, TP, and L-AP during the storage display at different temperature [**(A)** 4°C; **(B)** 20°C; **(C)** 50°C]. The color discrimination is to distinguish four groups including control test, TBHQ, TP, and L-AP.

Peroxide value (PV) was quantified by measuring the concentration of lipid peroxides, which is normally considered as the product of primary lipid oxidation (33). As shown in Figure 1, the three antioxidants (TBHQ, TP, L-AP), as well as storage period, have a significant (*p* < 0.05) effect on the PV of HPBF. PV increased significantly (*p* < 0.05) both in the control and treated samples during the storage. The increased PV value suggested that the number of lipid peroxides produced by lipid oxidative processes increased gradually with time. A distinct drop before 28 days may be due to the unstable decomposition of primary lipid oxidation products into shorter chain hydrocarbon (34).

In the initial stage of storage, the PV of the L-AP treatment group was lower than that of the control group at any storage temperatures, followed by the TBHQ treatment group and TP treatment group. There was no clear distinction between the control group and the TP treatment group. The results indicated that TBHQ, followed by L-AP, had an antioxidant effect on primary lipid oxidation products for HPBF during storage. According to the findings of Wang et al. (35), both TBHQ and L-AP showed a significant (*p* < 0.05) antioxidant effect, which effectively retarded the oxidative degradation of polyunsaturated fatty acids. However, the antioxidant activity of TBHQ was better than that of L-AP in oil and fat, which was different from our results. This could be as a consequence that L-AP with compatible properties had a better antioxidant activity than TBHQ for a water-oil mixture of the HPBF. Besides, the antioxidant effect of TP was lower than TBHQ. A similar effect of TBHQ and TP was observed by Rababah et al. (36) who confirmed that TBHQ was the most effective in preventing lipid oxidation in cooked chicken, followed by TP. Although TBHQ had a higher lipid solubility than TP and L-AP, it was clear that L-AP had the best antioxidant impact on primary oxidation products of HPBF among three antioxidants in the study, followed by TBHQ.

#### Thiobarbituric acid reactive substance value

Thiobarbituric acid reactive substance are used as an estimate of the concentration of secondary lipid oxidation products and the results of TBARS are shown in Supplementary Figures 1D–F for HPBF with different treatments (TBHQ, TP, L-AP). As shown in Supplementary Figures 1D–F, TBARS values of HPBF are significantly different between storage temperatures during storage. TBARS values increased faster at a higher temperature (50°C) compared to that at lower temperature (4 and 20°C), which was consistent with the production of primary lipid oxidation (PV value) as mentioned above. Interestingly, TBARS values gradually increased during the first 14 days at 50°C and then decreased during the following stages. The decrease of TBARS values with time was attributed to the advanced reactions of secondary lipid oxidation products with protein residues (33). The result of TBARS increased and then decreased during storage, which have previously been reported by Koutina et al. (33) who explained that interesting observations were a consequence of secondary lipid oxidation products being consumed by proteins to produce oxidized modified proteins.

In contrast to our suspicion, there were no variations in lipid oxidation (*p* > 0.05) among the three control treatments at any storage temperatures. It demonstrated that the three antioxidants had a limited ability to inhibit secondary lipid oxidation products in HPBF during the storage. A similar finding was reported by Jin et al. (37) who affirmed that TBHQ or L-AP alone did not show many protective properties on heat-treated corn oil with the TBARS assay.

### 1,1-Diphenyl-2-picrylhydrazyl scavenging activity

**FIGURE 2:**
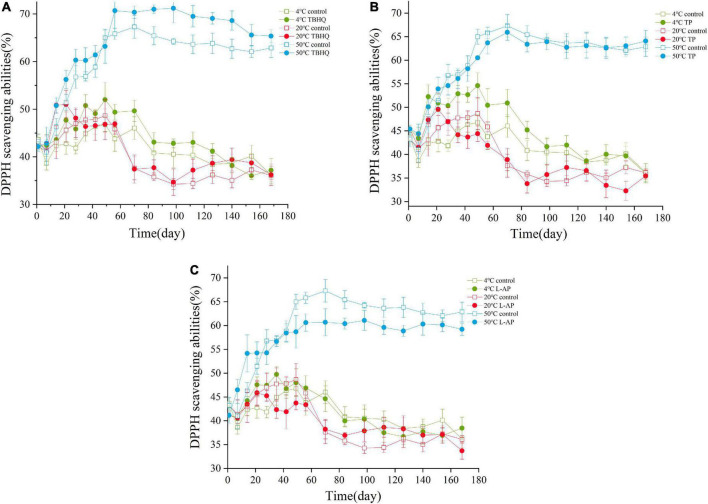
Levels of DPPH during the storage of HPBF with the addition of TBHQ **(A)**, TP **(B)**, and L-AP **(C)**. The discrimination of the shape is shown to distinguish the control group and the treatment groups. The color discrimination represents various temperature (4, 20, and 50°C).

The assay of DPPH scavenging activity is a simple method to assess the ability of antioxidants to trap free radicals (38). The DPPH scavenging activity is related to the delocalization of the unpaired electron throughout the molecule, such as peroxide-free radical, hydroxyl radicals and reactive oxygen species (38). The antioxidant effectiveness of TBHQ, TP, and L-AP evaluated in HPBF during the storage are shown in Figure 2. Based on the results in Figure 2, the DPPH scavenging ability increased first and then decreased during storage. One unanticipated discovery was that the values of DPPH scavenging ability were always the largest in the storage condition of 50°C. This was mainly owing to the ease with the Maillard reaction that occurs as the temperature rises. The Maillard reaction products had an effective antioxidant effect, which resulted in enhanced DPPH scavenging ability through the conversion of DPPH into a stable DPPH-H form (39, 40). The values gradually decreased and tended to be flat as storage time increased indicating that the oxidation reaction of HPBF was caused by the consumption of antioxidants. These findings would be confirmed by the results of oxidative stability analysis.

Compared with the control group, the addition of TBHQ, TP, and L-AP in HPBF could efficiently scavenge DPPH radicals and inhibit oil oxidation after being stored at a low temperature (4°C) for a short period. There was no significant difference in free radical scavenging between the three treatment groups at room temperature (20°C). TBHQ had an effective DPPH scavenging ability at higher temperatures (50°C), which indicted that its antioxidant impact was substantially greater than that of TP and L-AP. The results were consistent with previous research findings reported by Jin (41). TBHQ as one outstanding synthetic antioxidant reported by Liang et al. (42) who found that TBHQ can effectively inhibit the formation of free radicals and hence contributes to the stabilization of lipids. More unexpectedly, HPBF with the addition of L-AP (as another synthetic antioxidant), exhibited a lower DPPH scavenging activity than that of the control group at a higher storage temperature (50°C). This could be associated with the initial acidity of HPBF or the stability and structure of L-AP. Under acidic conditions, L-AP could be entirely hydrolyzed to ascorbic acid and their respective fatty acids in the reporter of EFSA and ANS (43), which affect its own free radical scavenging ability. Comparatively, TBHQ had a noticeable effect on scavenging free radicals terminating the free radical chain reaction, which could be attributed to its molecular structure, high thermal resistance and stability.

### Evaluation of physicochemical profiles

#### Browning

Browning is one of the most common natural phenomena during the processing and storage of food (44). As shown in Supplementary Figures 1G–I, the brown color (A420) of samples increased over time, particularly when stored at 50°C; A420 values of samples stored at 50°C were higher compared to that of samples stored at 4 and 20°C at each time point during storage. The results suggested that Maillard browning depended greatly on temperature and time. However, it was worth noting that the growing trend of browning in the three treatment groups (TBHQ, TP, and L-AP) did not differ substantially. The similarity of A420 values between TBHQ, TP, and L-AP groups indicated that the addition of antioxidants had little effect on the browning of HPBF during storage. In fact, products of the Maillard browning reaction during storage were also regarded as antioxidants and could interfere with the antioxidant effect from TBHQ, TP, and L-AP (45, 46). For example, Mshayisa (47) revealed that Maillard reaction products showed higher antioxidant capacity than TBHQ (0.02% w/w) according to the TBARs assay in a glucose-casein model system. As also demonstrated by Kirigaya et al. (48), melanoidin pigment, a component of Maillard browning, played an important role in the antioxidant activity. Accordingly, it may be suspected that the impact of three antioxidants on browning was much lower than that of Maillard reaction products during the storage.

#### pH

An examination of the acidity of HPBF with three antioxidants (TBHQ, TP, L-AP) treatment during storage (Supplementary Figures 2A–C) indicated that TBHQ, TP and L-AP did not alter (*p* > 0.05) the acidity values, except after 112 days during the low temperature (4°C) storage. TBHQ, TP, and L-AP may be able to limit the hydrolytic and oxidative rancidity of lipid to a certain extent in the long term at low temperatures. However, the activities of three antioxidants to control the change of acidity value was not significantly different (*p* > 0.05).

#### Water activity

Water activity (a_*w*_) is another important parameter to evaluate the physicochemical quality that governs storage stability (49). The effects of three antioxidants (TBHQ, TP, L-AP) on HPBF are shown in Supplementary Figures 2D–F. Based on the results, the a_*w*_ values of the TBHQ and L-AP groups were similar at all temperatures. The a_*w*_ of the HPBF samples in either TBHQ group or L-AP group equilibrated to the control indicating that the addition TBHQ or L-AP had little effect on the a_*w*_ of HPBF during storage. However, the TP group exhibited higher a_*w*_ values than other treatment groups and the control at both low (4°C) and room (20°C) temperature after 14 days of storage. No distinguished difference in a_*w*_ was found between the three treatment groups and the control at 50°C. The HPBF samples with the addition of TP or stored at 50°C may increase the value of a_*w*_, which may adversely affect the physicochemical profiles and stability of the HPBF samples.

### Analysis of volatility odorants

**FIGURE 3:**
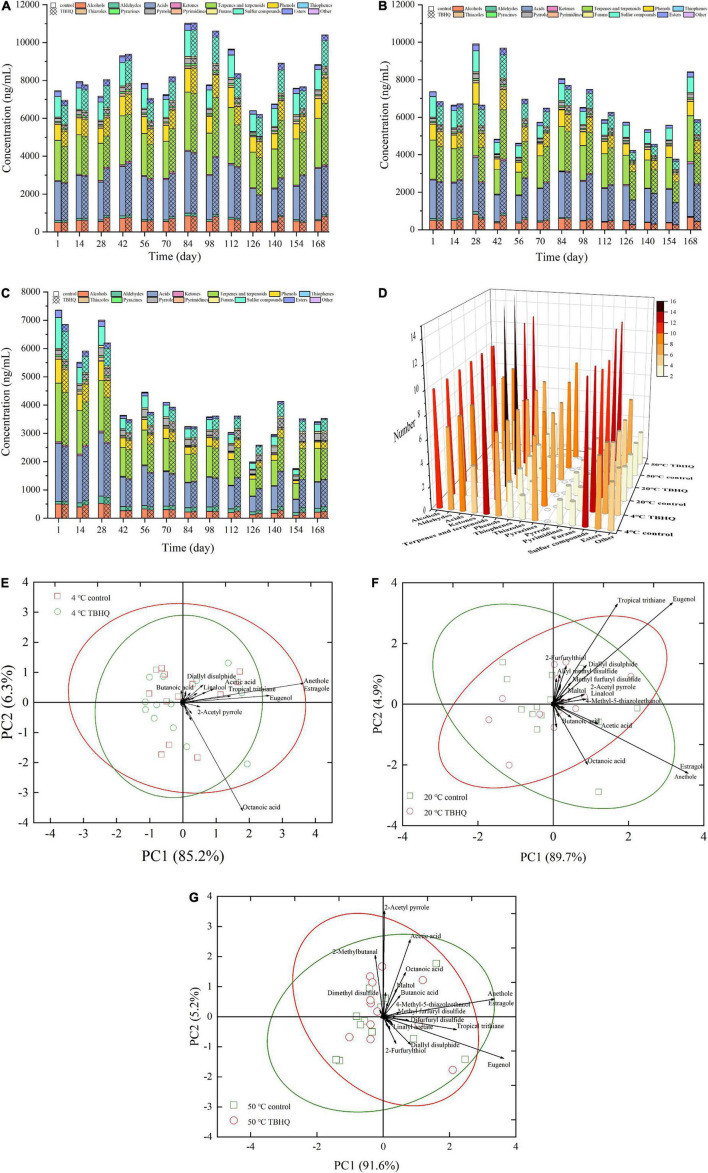
Effect of TBHQ on the quantity [**(A)** 4°C; **(B)** 20°C; **(C)** 50°C] and concentration **(D)** of volatile odorants in HPBF during storage. PCA diagram shows the difference of volatile odorants between TBHQ group and control test group [**(E)** 4°C; **(F)** 20°C; **(G)** 50°C].

Lipid oxidation rancidity affects flavor quality. It was attributed to the results that TBHQ had a better effect on controlling lipid oxidation than TP and L-AP, based on the evaluation of lipid oxidation stability and physicochemical profiles. The results of volatile odorants detected by SPME-GC-MS only discussed the effect of TBHQ on flavor profiles during storage. Volatile flavor compounds in HPBF were detected during storage (Table 1). As shown in Table 1, alcohols, terpenes, and terpenoids, nitrogenous heterocyclic compounds (thiophenes, thiazoles, pyrazines, pyrrole, and pyrimidines) and sulfur compounds accounted for a large percentage of volatility odorants. The concentration and amount of volatile odorants in HPBF altered with the storage environment, which was depicted in Figures 3A–D. The concentration of volatile components in HPBF did not exhibit significant changes at different temperatures (4 and 20°C). All of alcohols, acids, and sulfur compounds were significantly decreasing, when HPBF is held at 50°C. The accelerate experiment at the high temperature could give a negative impact on the total concentration of volatile chemicals. Interestingly, the concentration of aldehydes and furans in HPBF, and the number of pyrazines and sulfur compounds increased slightly with the storage time.

**TABLE 1:** Volatile odorants of HPBF identified by SPME-GC-MS/GC-O stored for 168 days.

No.	Compounds	Formula	CAS#*^a^*	RI*^b^* (DB-WAX)	Identification*^c^*
**Alcohols**					
1	Allyl alcohol	C_3_H_6_O	107-18-6	1109	RI,MS,O
2	1-Butanol	C_4_H_10_O	71-36-3	1142	RI,MS
3	Eucalyptol	C_10_H_18_O	470-82-6	1196	RI,MS,O
4	2-Methyl-1-butanol	C_5_H_12_O	137-32-6	1208	RI,MS
5	Hydroxyacetone	C_3_H_6_O_2_	116-09-6	1289	RI,MS,O
6	Linalool	C_10_H_18_O	78-70-6	1552	RI,MS,O
7	Propylene Glycol	C_3_H_8_O_2_	57-55-6	1593	RI,MS
8	(-)-Terpinen-4-ol	C_10_H_18_O	20126-76-5	1597	RI,MS
9	β-Acorenol	C_15_H_26_O	28400-11-5	1690	RI,MS
10	L-α-Terpineol	C_10_H_18_O	10482-56-1	1694	RI,MS
11	Phenylethyl Alcohol	C_8_H_10_O	60-12-8	1901	RI,MS,O
12	Maltol	C_6_H_6_O_3_	118-71-8	1950	RI,MS,O
13	α-Cadinol	C_15_H_26_O	481-34-5	2175	RI,MS
**Aldehydes**					
14	2-Methylbutanal	C_5_H_10_O	96-17-3	859	RI,MS,O
15	3-Hydroxybutanal	C_4_H_8_O_2_	107-89-1	1027	MS
16	Octanal	C_8_H_16_O	124-13-0	1282	RI,MS,O
17	2-Isopropyl-5-methylhex-2-enal	C_10_H_18_O	35158-25-9	1352	RI,MS
18	Non-anal	C_9_H_18_O	124-19-6	1388	RI,MS,O
19	Furfural	C_5_H_4_O_2_	98-01-1	1454	RI,MS,O
20	Benzaldehyde	C_7_H_6_O	100-52-7	1506	RI,MS,O
21	5-Methyl furfural	C_6_H_6_O_2_	620-02-0	1560	RI,MS,O
22	4-(1-Methylethyl)-benzaldehyde	C_10_H_12_O	122-03-2	1762	RI,MS
23	Tetradecanal	C_14_H_28_O	124-25-4	1867	RI,MS,O
24	10-Octadecenal	C_18_H_34_O	56554-92-8	1872	RI,MS
**Acids**					
25	Acetic acid	C_2_H_4_O_2_	64-19-7	1440	RI,MS,O
26	Propanoic acid	C_3_H_6_O_2_	79-09-4	1534	RI,MS,O
27	Butanoic acid	C_4_H_8_O_2_	107-92-6	1623	RI,MS,O
28	4-Methyl-pentanoic acid	C_6_H_12_O_2_	646-07-1	1800	RI,MS,O
29	Octanoic acid	C_8_H_16_O_2_	124-07-2	2058	RI,MS,O
30	Decanoic acid	C_10_H_20_O_2_	334-48-5	2271	RI,MS,O
31	4-oxo-Pentanoic acid	C_5_H_8_O_3_	123-76-2	2311	RI,MS
32	Sorbic Acid	C6H8O2	110-44-1	2120	RI,MS
**Ketones**					
33	4-Methyl-3-penten-2-one	C_6_H_10_O	141-79-7	1118	RI,MS
34	6-Methyl-5-hepten-2-one	C_8_H_14_O	110-93-0	1332	RI,MS,O
35	2(5H)-Furanone	C_4_H_4_O_2_	497-23-4	1730	RI,MS
36	Isosafrole	C_10_H_10_O_2_	120-58-1	1861	MS
37	Furaneol	C_6_H_8_O_3_	3658-77-3	2025	RI,MS,O
**Terpenes and terpenoids**					
38	(+)-α-Pinene	C_10_H_16_	7785-70-8	1016	RI,MS
39	D-Limonene	C_10_H_16_	5989-27-5	1186	MS
40	*trans*-β-Ocimene	C_10_H_16_	3779-61-1	1229	RI,MS
41	γ-Terpinene	C_10_H_16_	99-85-4	1236	RI,MS
42	3-Carene	C_10_H_16_	13466-78-9	1246	RI,MS
43	Terpinolene	C_10_H_16_	586-62-9	1272	RI,MS
44	α-Copaene	C_15_H_24_	3856-25-5	1480	RI,MS,O
45	1-Caryophyllene	C_15_H_24_	87-44-5	1582	RI,MS
46	Humulene	C_15_H_24_	6753-98-6	1653	RI,MS
47	Estragole	C_10_H_12_O	140-67-0	1661	RI,MS,O
48	γ-Muurolene	C_15_H_24_	30021-74-0	1680	RI,MS
49	α-Amorphene	C_15_H_24_	483-75-0	1717	RI,MS
50	Di-epi-α-cedrene	C_15_H_24_	50894-66-1	1718	MS
51	β-Bisabolene	C_15_H_24_	495-61-4	1724	RI,MS
52	δ-Cadinene	C_15_H_24_	483-76-1	1752	RI,MS
53	α-Curcumene	C_15_H_22_	644-30-4	1772	RI,MS
54	Anethole	C_10_H_12_O	104-46-1	1825	RI,MS,O
55	Calamenene	C_15_H_22_	483-77-2	1830	RI,MS
56	Elemicin	C_12_H_16_O_3_	487-11-6	2221	RI,MS,O
57	Myristicin	C_11_H_12_O_3_	607-91-0	2247	RI,MS
**Phenols**					
58	Phenol	C_6_H_6_O	108-95-2	1996	RI,MS
59	Methyleugenol	C_11_H_14_O_2_	93-15-2	2006	RI,MS
60	4-Ethyl-2-methoxy-phenol	C_9_H_12_O_2_	2785-89-9	2018	RI,MS
61	Eugenol	C_10_H_12_O_2_	97-53-0	2156	RI,MS,O
62	Isoeugenol	C_10_H_12_O_2_	97-54-1	2243	RI,MS
63	*trans*-Isoeugenol	C_10_H_12_O_2_	5932-68-3	2332	RI,MS
64	4-(2-Propenyl)-phenol	C_9_H_10_O	501-92-8	2328	RI,MS
**Thiophenes**					
65	3-Methylthiophene	C_5_H_6_S	616-44-4	1067	RI,MS
66	2-Methylthiophene	C_5_H_6_S	554-14-3	1068	RI,MS
67	2-Thiophenemethanol	C_5_H_6_OS	636-72-6	1934	RI,MS,O
**Thiazoles**					
68	2-Acetylthiazole	C_5_H_5_NOS	24295-03-2	1633	RI,MS
69	4-Methyl-5-thiazoleethanol	C_6_H_9_NOS	137-00-8	2296	RI,MS,O
**Pyrazines**					
70	2-Methylpyrazine	C_5_H_6_N_2_	109-08-0	1256	RI,MS
71	2,5-Dimethylpyrazine	C_6_H_8_N_2_	123-32-0	1314	RI,MS,O
72	2,6-Dimethylpyrazine	C_6_H_8_N_2_	108-50-9	1321	RI,MS,O
73	2-Ethylpyrazine	C_6_H_8_N_2_	13925-00-3	1326	RI,MS
74	2,3-Dimethylpyrazine	C_6_H_8_N_2_	5910-89-4	1338	RI,MS,O
75	2-Ethyl-6-methylpyrazine	C_7_H_10_N_2_	13925-03-6	1379	RI,MS,O
76	2,3,5-Trimethylpyrazine	C_7_H_10_N_2_	14667-55-1	1397	RI,MS,O
77	2-Methyl-3-propylpyrazine	C_8_H_12_N_2_	15986-80-8	1460	RI,MS
78	Acetylpyrazine	C_6_H_6_N_2_O	22047-25-2	1612	RI,MS,O
**Pyrrole**					
79	2-Acetyl pyrrole	C_6_H_7_NO	1072-83-9	1959	RI,MS,O
**Pyrimidines**					
80	2-Methylpyrimidine	C_5_H_6_N_2_	5053-43-0	1256	RI,MS
81	4-Methylpyrimidine	C_5_H_6_N_2_	3438-46-8	1256	RI,MS
**Furans**					
82	2-Pentylfuran	C_9_H_14_O	3777-69-3	1225	RI,MS
83	2-Acetylfuran	C_6_H_6_O_2_	1192-62-7	1494	RI,MS,O
**Sulfur compounds**					
84	Dimethyl disulfide	C_2_H_6_S_2_	624-92-0	1049	RI,MS,O
85	Diallyl sulfide	C_6_H_10_S	592-88-1	1131	RI,MS,O
86	Methyl propyl disulfide	C_4_H_10_S_2_	2179-60-4	1220	RI,MS,O
87	Allyl methyl disulfide	C_4_H_8_S_2_	2179-58-0	1270	RI,MS,O
88	2-Methyl-3-furanthiol	C_5_H_6_OS	28588-74-1	1304	RI,MS,O
89	Dimethyl trisulfide	C_2_H_6_S_3_	3658-80-8	1363	RI,MS,O
90	Dipropyl disulfide	C_6_H_14_S_2_	629-19-6	1369	RI,MS,O
91	2-Furfurylthiol	C_5_H_6_OS	98-02-2	1426	RI,MS,O
92	Diallyl disulfide	C_6_H_10_S_2_	2179-57-9	1470	RI,MS,O
93	Methyl allyl trisulfide	C_4_H_8_S_3_	34135-85-8	1574	RI,MS,O
94	Tropical trithiane	C_9_H_18_S_3_	828-26-2	1712	RI,MS,O
95	Methyl furfuryl disulfide	C_6_H_8_OS_2_	57500-00-2	1791	RI,MS,O
96	Furfuryl sulfide	C_10_H_10_O_2_S	13678-67-6	2045	RI,MS,O
97	Difurfuryl disulfide	C_10_H_10_O_2_S_2_	4437-20-1	2546	RI,MS,O
**Esters**					
98	Ethyl octanoate	C_10_H_20_O_2_	106-32-1	1435	RI,MS
99	Linalyl acetate	C_12_H_20_O_2_	115-95-7	1556	RI,MS,O
100	Ethyl decanoate	C_12_H_24_O_2_	110-38-3	1643	RI,MS
101	Terpinyl acetate	C_12_H_20_O_2_	80-26-2	1690	RI,MS
102	Methyl salicylate	C_8_H_8_O_3_	119-36-8	1756	RI,MS
103	Eugenyl acetate	C_12_H_14_O_3_	93-28-7	2246	RI,MS
**Other**					
104	*o*-Cymene	C_10_H_14_	527-84-4	1259	RI,MS
105	1-Methyl-3-(1-methylethyl)-benzene	C_10_H_14_	535-77-3	1257	RI,MS
106	2,2′-Methylenebis furan	C_9_H_8_O_2_	1197-40-6	1603	RI,MS
107	Butylated hydroxytoluene	C_15_H_24_O	128-37-0	1902	RI,MS

^a^CAS, Chemical Abstract Service registration number.

^b^RI, Retention indices calculated using the n-alkanes of C7-C40 on DB-WAX column.

^c^MS, mass spectrometry; O, reference standard odor description.

The decrease of alcohols and the increase of aldehydes were reported to be due to oxidation reactions under high temperature conditions, which promoted the conversion of alcohols to aldehydes, or the generation of aldehydes attributed to lipid oxidation or thermal degradation of amino acids (50). As reported by Frank et al. (51), the increase of 2-methylbutanal was produced through thermal Strecker degradation of isoleucine. The protein oxidation and Maillard reaction were responsible for Strecker aldehydes have been reported by Zhou et al. (52) and Wen et al. (53). However, alcohols and aldehydes in the TBHQ treatment group were higher than those in the control group as storage time went on, indicating that the antioxidant impact of TBHQ was able to stabilize their reaction activity throughout storage.

Pyrazines are usually produced at higher temperatures and contribute to the roast and nutty aroma of overall flavor attributes (54). Changes in concentration and amount during storage may due to the decomposition of proteins and amino acids (55) or the generation of Maillard reaction *via* sugar dehydration or fragmentation (56), instead of lipid oxidation. This confirmed that TBHQ had no significant effect on pyrazines.

Sulfur compounds are considered to be essential volatile aroma active compounds due to their lower odor threshold (57). The decrease or increase of sulfur compounds had a direct effect on the overall flavor profiles of HPBF during storage. Some sulfur compounds include 2-methyl-3-furanthiol, dipropyl disulfide, 2-furfurylthiol, diallyl disulfide, tropical trithiane, methyl furfuryl disulfide, and difurfuryl disulfide decreased over the time. The reaction of lipid oxidation degradation products with H_2_S produced by Strecker degradation of cysteine, interfering with the reaction pathway and reducing the reactants of sulfur compounds, which resulted in a large reduction in the overall content of sulfur compounds (54). Thiols and other sulfur compounds with oxygen heterocycles were extremely reactive, susceptible to oxidation, and easily affected by the environment (58). They can degrade thermally to produce a variety of carbonyl and hydroxyl carbonyl components (59). However, an addition of TBHQ increased the total concentration of sulfur compounds during storage, which could attribute to that TBHQ was more effective in inhibiting lipid oxidation and maintaining greater oxidation stability of HPBF than control group. Notably, in the TBHQ treatment group and the control group, dimethyl disulfide and dimethyl trisulfide increased in the later stage of storage and accounted for a large proportion. These volatiles had been previously linked to meat spoilage and strong objectionable odors (51, 60).

Furans as oxygen-containing heterocyclics are major intermediate products of the Maillard reaction, mostly from sugar degradation (61). It had little correlation with fat oxidation during storage. The concentration of 2-acetylfuran (one of the oxygenated heterocyclic compounds) gradually increased with storage time. However, this odorant with a high odor threshold had little effect on the flavor profiles (62).

Generally speaking, only a few volatile components were important aroma active compounds, which contributed effectively to the overall flavor properties. A total of 51 odorants were sniffed and semi-quantified from the headspace of the HPBF during the storage (Table 1). In order to more accurately and intuitively analyze the influence of the TBHQ treatment group on volatile aroma components in HPBF, PCA applied to reveal patterns in the dataset was shown in Figures 3E–G. At 4, 20, and 50°C, the Bi-plot of the sum of first two principal components accounted for 91.5, 94.6, and 96.8% of the total variance in raw data, respectively, which explained fully the variation trend of the volatile odorants. The data points with distinct markers belonged to different treatments, according to the distribution of data mapped on the principal components. There was a hazy demarcation between HPBF samples from the blank and TBHQ treatment groups during the storage at any temperature. The importance of different volatile odorants to HPBF with different treatments could be distinguished by the visual loading matrix (the black lines). Closer inspection of Figures 3E–G revealed that clove-smelling eugenol, anise-smelling anethole, and estragole had a higher contribution value to the overall flavor attribute of HPFB during storage due to their positions far from the coordinate origin. The loading matrix of 2-furfurylthiol, diallyl disulfide, tropical trithiane, and methyl furfuryl disulfide increased were larger than that of control group with the increasing of temperature at 4 and 20°C, implying that these odorants have a higher contribution to the flavor profiles of TBHQ treatment group. 2-Methylbutanal, 2-acetyl pyrrole, and difurfuryl disulfide were more prominent in the TBHQ treatment group at 50°C. This was possible because TBHQ could maintain the oxidation stability of HPBF during the storage, owing to the antioxidant effect. The loss of some active aroma components (2-methylbutanal, 2-methyl-3-furanthiol, 2-furfurylthiol, diallyl disulfide, tropical trithiane, methyl furfuryl disulfide, and difurfuryl disulfide) in HPBF during storage was minimized in the TBHQ treatment group. It was interesting to find that the contribution value of octanoic acid gradually decreases, indicating that it was oxidized due to the influence of storage temperature and time. In short, these findings provide vital information about that several volatile odorants were affected by TBHQ contributing to the overall flavor qualities during storage. Meanwhile, these odorants were the main differences between the blank and TBHQ treatment group.

## Conclusion

The effect of three antioxidants (TBHQ, TP, and L-AP) on the flavor profiles, oxidative stability and the physicochemical profiles in HPBF at different temperatures during the storage was analyzed. The results indicated that TBHQ, TP, and L-AP had a little influence on the browning, a_*w*_, and acidity of HPBF. Based on the results of an oxidative stability assessment, L-AP had a better ability to limit the production of primary oxidation products than TBHQ, although both L-AP and TBHQ exhibited poor control over secondary lipid oxidation products regardless of storage temperature. In particular, TBHQ performed better in the DPPH radical scavenging assays, inhibiting lipid oxidation during storage with a higher DPPH radical scavenging ability. According to the results of oxidative stability and physicochemical profiles, HPBF with the addition of TBHQ exhibited greater lipid stability than L-AP, followed by TP. In addition, TBHQ could inhibit high-activity compounds from being oxidized and forming other objectionable odorants. However, an addition antioxidant (i.e., TBHQ) had a lower impact on flavor qualities during storage than storage temperature and storage time. It was undeniable that TBHQ may play an antioxidant role in HPBF during storage. The developed compound additive containing TBHQ had the potential to be employed as an effective additive in a complex system of beef flavor with the capacity to postpone oxidation reactions thereby enhancing flavor quality.

## Data availability statement

The original contributions presented in this study are included in the article/Supplementary material, further inquiries can be directed to the corresponding authors.

## Author contributions

ZZ: data curation, methodology, writing – original draft preparation, investigation, and validation. FM: writing – review and editing and funding acquisition. BW: writing – review and editing, project administration, and funding acquisition. YC: conceptualization, supervision, and project administration. All authors contributed to the article and approved the submitted version.

## Abbreviations

HPBF: heat processed beef flavor
TBHQ: tert-butylhydroquinone
TP: tea polyphenol
L-AP: L-ascorbyl palmitate
BHA: butylated hydroxyanisole
ORP: oxidation reduction potential
PV: peroxide value
TBARS: thiobarbituric acid reactive substances
a_*w*_: water activity
SPME-GC/MS: solid phase microextraction-gas chromatography/mass spectrometry.
