# Supplementary material for: Effects of antioxidants on physicochemical properties and odorants in heat processed beef flavor and their antioxidant activity under different storage conditions
Source: Front Nutr. 2022 Aug 30;9:966697. doi: 10.3389/fnut.2022.966697 (PMC9468785; doi:10.3389/fnut.2022.966697)

Supplementary Material

Figures

Figure S1 Levels of ORP (**A**, **B**, **C**), TBARS (**D**, **E**, **F**) and A420 (**G**, **H**, **I**) values during the storage of HPBF with the addition of TBHQ (**A**, **D**, **G**), TP (**B**, **E**, **H**) and L-AP (**C**, **F**, **I**). The discrimination of the shape is shown to distinguish the control group and the treatment groups. The color discrimination represents various temperature (4 ℃, 20 ℃ and 50 ℃).

Figure S2 pH (**A**, **B**, **C**) and a_w (**D**, **E**, **F**) values of HPBF with the addition of TBHQ, TP and L-AP during the storage display at different temperature (4 ℃: **A**, **D**; 20 ℃: **B**, **E**; 50 ℃: **C**, **F**). The color discrimination is to distinguish four groups including control test, TBHQ, TP and L-AP.

Figure S1

A

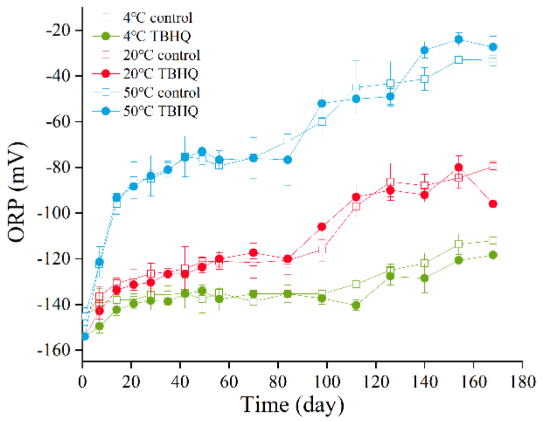

B

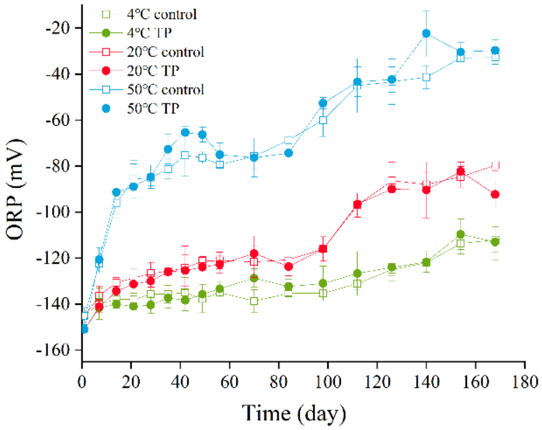

C

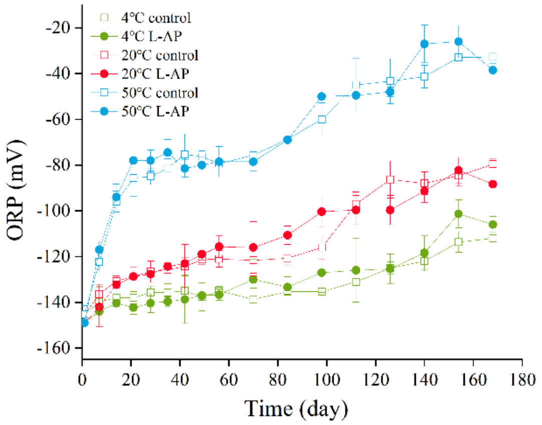

D

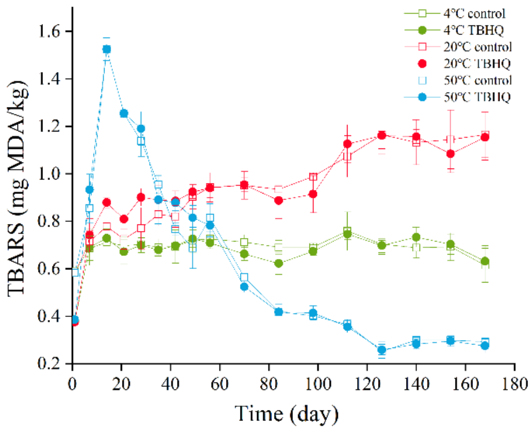

E

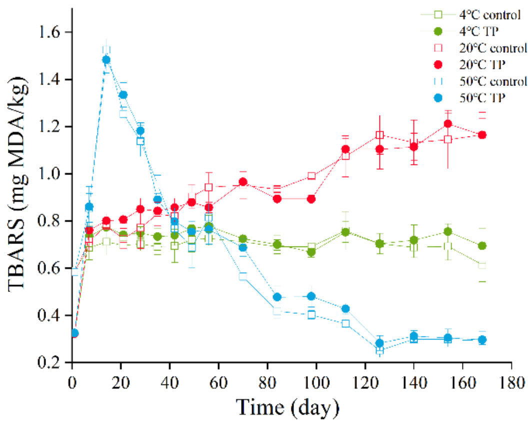

F

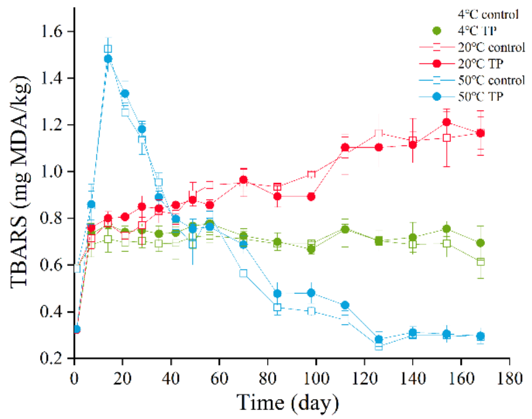

G

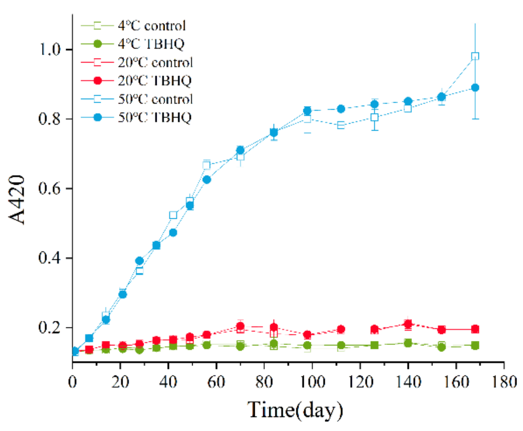
4°C control
4°C TBHQ
20°C control
20°C TBHQ
50°C control
50°C TBHQ
A420
Time(day)

H

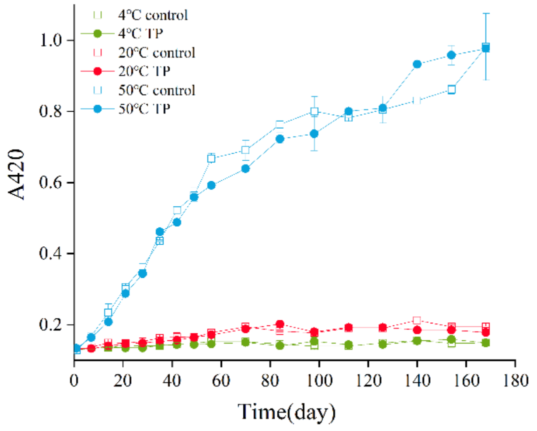
4°C control
4°C TP
20°C control
20°C TP
50°C control
50°C TP
A420
Time(day)

I

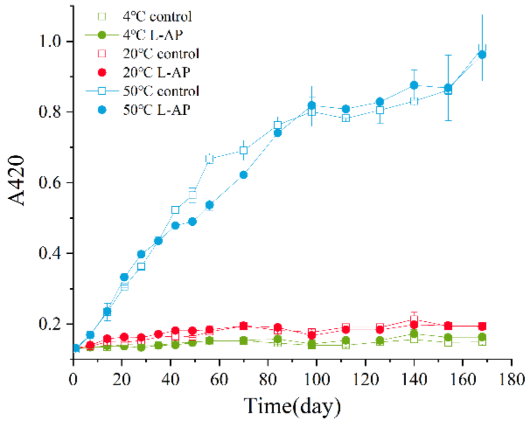
4°C control
4°C L-AP
20°C control
20°C L-AP
50°C control
50°C L-AP
A420
Time(day)

Figure S2

A

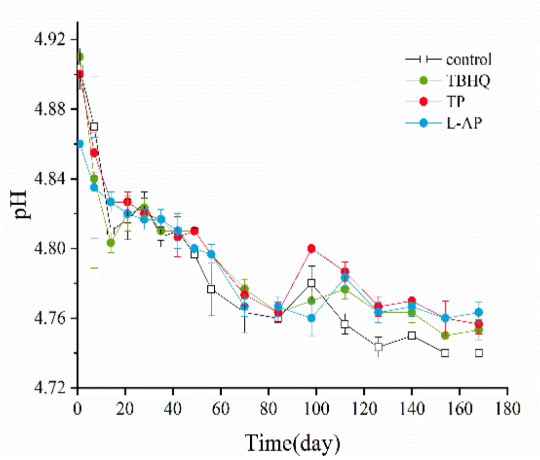

B

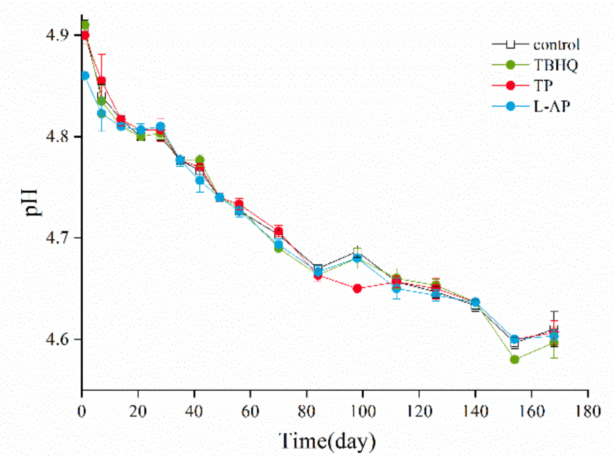

C

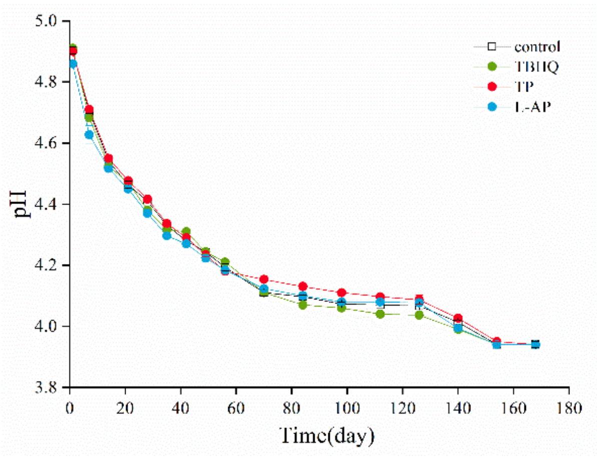

D

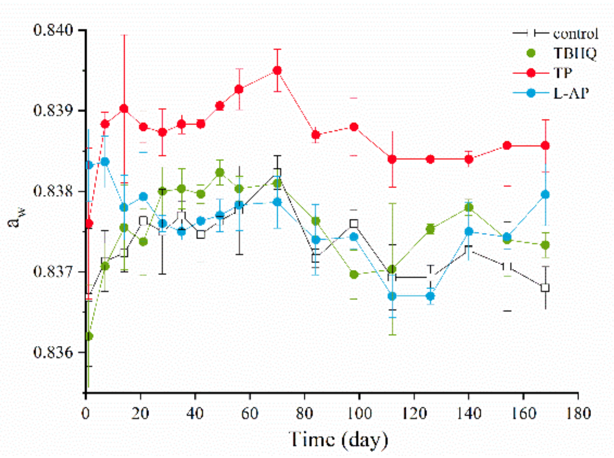

E

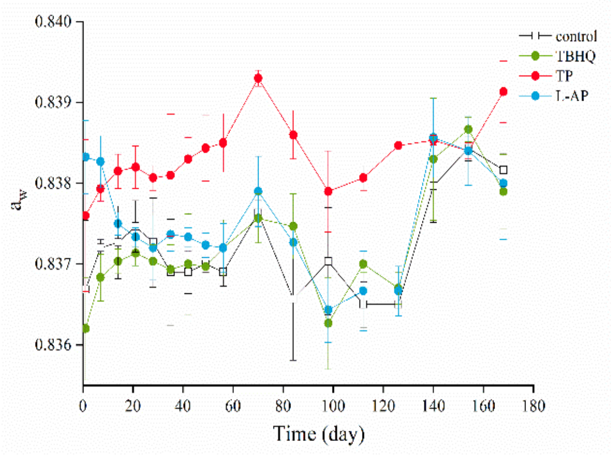

F

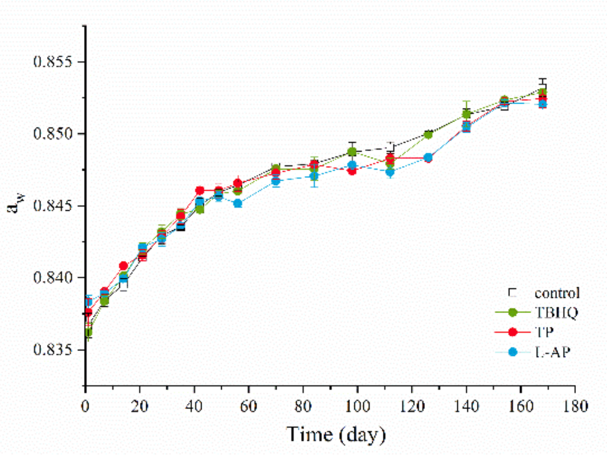

Supplement: Supplementary file 1 [file Data_Sheet_1.pdf]
